# A Modal Interpretation of Quantum Spins and Its Application to Freudian Theory

**DOI:** 10.3390/e24101419

**Published:** 2022-10-04

**Authors:** Giulia Battilotti, Miloš Borozan, Rosapia Lauro Grotto

**Affiliations:** 1 Department of Mathematics, University of Padua, Via Trieste, 63, 35122 Padova, Italy; 2 Department of Neurosciences, Imaging and Clinical Sciences, G. d’Annunzio University of Chieti-Pescara, Via dei Vestini, 31, 66100 Chieti, Italy; 3 Center for Advanced Studies and Technologies (C.A.S.T.), Via Luigi Polacchi, 11, 66100 Chieti, Italy; 4 Department of Health Sciences, University of Florence, Via San Salvi, 12, 50135 Firenze, Italy

**Keywords:** quantum states, uncertainty, metalanguage, infinite singletons, modality, negation, quantum cognition, psychoanalytic formal models

## Abstract

In the present paper, we aim to develop a formal quantum logic theory of the interplay between conscious and unconscious processes of the human mind, a goal that has already been envisaged in quantum cognition; in doing so, we will show how the interplay between formal language and metalanguage allows for characterizing pure quantum states as infinite singletons: in the case of the spin observable, we obtain an equation defining a modality that is then re-interpreted as an abstract projection operator. By including a temporal parameter in the equations and by defining a modal negative operator, we derive an intuitionistic-like negation, for which the non-contradiction law is seen as an equivalent of the quantum uncertainty. Building on the psychoanalytic theory of Bi-Logic by Matte Blanco, we use modalities in interpreting the emergence of conscious representations from an unconscious one, and we demonstrate that this description fits well with Freud’s view of the role of negation in mental processes. Psychoanalysis, where affect plays a prominent role in shaping not only conscious, but also unconscious representations, is therefore seen as a suitable model to expand the domain of quantum cognition to the broader field of **affective quantum cognition.**

## 1. Introduction

Classical physics was developed assuming the sharp distinguishability of objects. However, quantum physics subsequently discovered that the possibility of identifying and separating objects cannot be taken for granted. Still, at that point, the mathematical language for the “exact sciences” had been already developed, so its implicit assumptions were applied to quantum physics as well, in particular those concerning how to distinguish and characterise objects. Considering the relevance of the language for the scientific processes of theory genesis and model building [1], we start by exploring the epistemological and theoretical consequences and opportunities hidden in the crevices of the latter point. Namely, we begin by asking if indeed we can say that an object we need to describe is uniquely characterized by the language we are adopting. As we shall see, such question cannot entail a formal answer but a psychological one. Actually, it is the approach suggested by Federigo Enriques at the beginning of last century in [2], where he proposed the idea of a *psychological logic* rather than a formal one (in his words: “Anyway, we recognize that Logic can be regarded as a set of norms, which must be observed to obtain a coherence of thinking. On the other hand, this can also be expressed by saying that there are some mental procedures in which certain coherence conditions, that indeed are termed logical procedures, are willingly satisfied. *In this sense, Logic can be regarded as a part of Psychology.*”; [2], Chapter III “The problems of Logic”, Section 3 “Symbolic Logic and psychological Logic”, p. 164, italics in the text, translation by the authors). Historically, the formalist approach won, since it can more easily guarantee to accomplish Leibnitz’s motto *Calculemus!* that has been so important in the scientific development. In particular, the formal language adopted in mathematics, and then in physics, characterized as first order language since the 19th century, has been used without further discussions, even when Kurt Gödel, in his incompleteness theorems, pointed out its intrinsic limitations due to its inability to deal with the gap between the metalevel and the object level [3]. Indeed, we as human beings can overcome such a gap, since our mind deals with it informally. This implies that an important component of what is necessary to the construction of the scientific knowledge has been left out of the analysis by formal science itself. In turn, this implies a need to search for an integration of a component left without discussion up to now, in order to better accomplish Leibnitz’s *Calculemus!*. Since the ultimate responsibility for the not yet integrated component of our process of knowledge lies with mind, we believe that it is necessary to consider the point of view expressed in [2]. In our opinion, it has been widely neglected, or, at least not considered enough, by logicians, with the quite radical attitude it deserves in order to be effective. Then, an instance of a well-known paradox—digging deep into the heart of a single scientific discipline, one suddenly found themselves on the borders of another one—is witnessed [1,4]. Therefore, our work, devoted to an analysis of a way of describing quantum objects, is actually linked to the psychological roots of that method. Our references in psychology, are the foundational units of Freudian theory, the theory of representation [5] and Freud’s subsequent characterization of the primary process [6], together with Matte Blanco’s Bi-logic [7] that offers a unique opportunity of integration between psychoanalysis and logic. The final emphasis is on the mental aspect, since, in our view, it is the core for a better comprehension of the process of scientific knowledge that we pursue. As for theoretical physics, our analysis can show how the representation of quantum objects corresponds to mental representations, by which unavoidably we interpret nature. In the meantime, we also obtain an application of quantum physics to cognitive studies, on one side, and a new interpretation, in formal terms, in the framework of metapsychological studies, on the other.

Specifically, considering the issue of the characterisation of objects described above, and in order to see how an object can be uniquely characterized in the scientific language, one has to observe that the notion of *term*, in the first order language, confines the real distinction between constants and variables, both used to indicate objects, to the metalevel. This rules out the possibility of discussing the characterization of the objects *inside* the formal apparatus. This leads us to our first question—how can assertions on quantum states be related to constants and variables of first order language?

In an attempt to provide an answer, we start with a description of a model for the representation of quantum states, confined to the case of one observable only, developed in first order language and originally proposed in [8]. It is based on the formalisation of assertions derived from the informal ones contained in the axiomatization of quantum mechanics. The technique applied relies on the use of suitable equations that define the logical connectives, in the view of basic logic [9], taking into account the characterizations of the algebraic formalism. As stressed in [9], the technique of equations is suited to deal with the interplay between the metalevel and the object level. Furthermore, basic logic has an important role in proof theory for quantum logics; see [10]. More generally, it is an instance of a minimalist approach to the mathematical foundations; see [11]. In our work that pursues the integration of elements from psychoanalysis, a minimalist approach is recommended, since only basic assumptions and constructions can offer the way not to destroy the objects we need to model. Therefore, the approach obtained by adopting basic logic is suited in order to answer our question finding its psychological roots.

We focus our attention on the spin observable, and we discuss the notion of spin conceived as *infinite singleton*, a construct put forward in [8]. Subsequently, we introduce a modality, interpreted as an abstract form of projector, and show that it coincides with the modal operator of *S*4. This result supports our search for two reasons: on one side, it corresponds to the findings by Gödel [12], who proposed such a modal operator as a way to overcome the incompleteness (created by the finitary notion of provability formalizable in first order arithmetic), by adopting an infinitary notion of provability, represented by the modality itself. The difference is that now the modality is obtained from qubits, namely from considering the whole Bloch sphere, rather than considering a couple of poles only, namely the couple of bits true/false of propositional classical logic, to which the modality itself is added to form the system *S*4. On the other side, the result corresponds to the findings of [13] that characterize intuitionistic logic (equivalent to *S*4), as the logic of quantum registers measurement. Additionally, we show that the introduction of a temporal parameter allows a finite reading of the modality. Hence, its interpretation can be split into two cases: assertion, corresponding to the finite reading of the modal operator itself, and negation, originated as an “abstract anti-projector”. Finally, this allows for establishing of the correspondence between quantum uncertainty, on one hand, and the law of non-contradiction in logic on the other.

In the second part of the paper, we use these theoretical advancements to put forward a novel modelization of Freudian theory of the mind. The effort to formalize Freudian theory dates back to the pioneering work of A. Khrennikov, who was the first to recognize in Freudian Unconscious (as described in the Interpretation of Dreams [6]) a well-defined topological structure that is the ultrametric space based on p-adic numbers (see [14,15], as well as the monograph [16]).

Along with this line of interest, our proposal is still founded on the pivotal constructs of classical psychoanalysis, namely the characterization of the primary process from The Interpretation of Dreams [6]; furthermore, we explore the consequences of the theory of representations, already developed during Freud’s *neurological* period [5]. The theory of representations enlightens the crucial role of language in regulating access of Unconscious representations to consciousness, therefore suggesting the potential relevance of logic as a formalization framework for psychoanalytic theory. Then, we also build on the logical reformulation of the Freudian Unconscious proposed by the Chilean psychoanalyst I. Matte Blanco [7]. Namely, he described the structural Unconscious proposed by Freud in terms of two logical principles, the Principle of Symmetry and the Principle of Generalisation. The theoretical construction based on these two principles manifests remarkable explanatory capabilities and has led to a new conceptualization of the the objects of the Unconscious in terms of *infinite sets* [7]. Matte Blanco’s Bi-logic model has been hence reinterpreted by means of infinite singletons [17] which, among else, has made it a suitable theoretical platform for a reconsideration of the original Freudian idea in terms of quantum spins. As we shall see, this approach actually allows also for a fruitful discussion of the border between infinite and finite, in terms of the pioneering Freudian description in terms of presence/absence of linguistic representations. In addition, the introduction of parameter of time in the theoretical conception further widens the perimeter of explanation to include both negation and non-contradiction. Then, we find a correspondence with the characterization of mental processes as primary and secondary, introduced in the Interpretation of Dreams. Moreover, as suggested in [17], the theory of Representations finds a correspondence with infinite singletons too. Finally, we put forward a reinterpretation of a famous clinical example from Freud’s paper *Negation* [18], based on the idea of *negation as finitization*.

## 2. Describing the State of a Particle with Respect to a Given Observable by First Order Formulae

As is well known, the state of a quantum particle, represented in a Hilbert space, can be characterized as a projector, namely as a density operator of rank 1. A quantum measurement makes the pure state collapse into a mixture, whose density matrix, given an orthonormal basis, can be represented as a convex combination of its projectors, where the interference terms are 0.

### 2.1. Representation of Pure and Mixed States

The approach developed in [8] and previous papers characterizes the pure state and the corresponding mixture after measurement by the formalization of the meta-linguistic assertions concerning the state of the particle. Convenient equations to define connectives, in the view of basic logic [9], are adopted. Such an approach is revised below and then applied throughout the paper.

Let us consider a particle A. The measurement of A, considering a certain observable, gives a set of actual outcomes

$$D_{Z}=\left\{\left(s_{n},p\left\{Z=s_{n}\right\}\right):n\in I\right\}$$

where *Z* is the random variable associated with the measurement, whose outcomes are the states $s_{n}$, considered with their frequencies $p\{Z=s_{n}\}$, where $p\{Z=s_{n}\}>0$. In particular, when there is a unique certain outcome *s*, $D_{Z}$ is a singleton: {u}, where u=(s,1). In general, if Γ is a set of assumptions for the measurement of particle A, one can say that:

$$\Gamma \;\text{yields}\;A\;\mathrm{is}\;\mathrm{found}\;\mathrm{in}\;\mathrm{state}\;s_{n}\;\mathrm{with}\;\mathrm{probability}\;p\{Z=s_{n}\}.$$

Let us write *yields* by the sequent sign for consequence: ⊢, and the formula “A is found in state $s_{n}$ with probability $p\{Z=s_{n}\}$” by $A(t_{n})$, $t_{n}$ a closed term of the language for $\left(s_{n},p\left\{Z=s_{n}\right\}\right)$. Then,

$$\Gamma ⊢A(t_{n})$$

for all *n*. In order to deal with such assertions, we can:

- group all the assertions at the meta-level, adopting the meta-linguistic link *forall*, and say
  $$\Gamma ⊢A\left(t_{i}\right)\;forall\;t_{i}\in D_{Z}$$
- introduce a variable *z* of the language and import the assumptions $t_{i}\in D_{Z}$ as a formal premise $z\in D_{Z}$, considering the overall assertion
  $$\Gamma ,z\in D_{Z}⊢A(z)$$

In the first case, the equation:

(1) $$\Gamma ⊢\left(\forall_{\omega }t_{n}\in D_{Z}\right)A\left(t_{n}\right)\;if\;\;and\;\;only\;\;if\;\Gamma ⊢A\left(t_{n}\right)\;forall\;t_{n}\in D_{Z}$$

defines a connective $\forall_{\omega }$ which can group all the assertions $A(t_{n})$, where the terms $t_{n}$ are parameters. The formula

$$\left(\forall_{\omega }t_{n}\in D_{Z}\right)A\left(t_{n}\right)$$

describes the overall result of a measurement, namely the mixed state after measurement. When the result of the measurement is certain, namely the set $D_{Z}$ is a singleton {u}, where u=(s,1), $\left(\forall_{\omega }t_{n}\in D_{Z}\right)A\left(t_{n}\right)$ is the propositional formula A(u).

In the second case, the equation:

(2) $$\Gamma ⊢\left(\forall x\in D_{Z}\right)A\left(x\right)\;if\;and\;only\;if\;\Gamma ,z\in D_{Z}⊢A\left(z\right)$$

defines the usual universal quantifier ∀ [11]. The pure state of the particle, with respect to the given observable only, is described by the universal proposition

$$\left(\forall x\in D_{Z}\right)A\left(x\right)$$

that is, considering the variable in the formal object language instead of the parameters is a logical way to add the presence of interference. When $D_{Z}=\left\{u\right\}$ is a singleton, the universal formula $\left(\forall x\in D_{Z}\right)A\left(x\right)$ is equivalent to A(u) and hence the representation of the pure state coincides with the representation of the result of measurement. We shall interpret this point in the following.

We see now that, in general, this is not the case and hence that the interference can be represented by the presence of the variable inside the object language. The formulae $\left(\forall_{\omega }t_{n}\in D_{Z}\right)A\left(t_{n}\right)$ and $\left(\forall x\in D_{Z}\right)A\left(x\right)$ in general are not equivalent, otherwise one would go against Gödel’s first incompleteness theorem (for, if Equation (1) were as strong as Equation (2), Gödel’s formal sentence asserting its own unprovability (that is, a universal formula for which every particular instance with respect to a numeral-natural parameter *n*-is provable) could be actually derived, then producing a contradiction). However, the following direction is derivable:

$$\left(\forall x\in D_{Z}\right)A\left(x\right)⊢\left(\forall_{\omega }t_{n}\in D_{Z}\right)A\left(t_{n}\right)$$

by substitution of the closed terms to the variable, see [8]. Thus, the consequence $\left(\forall x\in D_{Z}\right)A\left(x\right)⊢\left(\forall_{\omega }t_{n}\in D_{Z}\right)A\left(t_{n}\right)$ represents an irreversible collapse. In particular, instantiating the variable by the closed terms by substitution describes the measurement. We conclude that the presence of the variable at the object level makes the interference possible, since it acts as a glue joining the elements $t_{n}$, which do not interfere when considered as parameters at the metalevel. In summary, the universal proposition $\left(\forall x\in D_{Z}\right)A\left(x\right)$ can represent the pure state of the particle.

We need to stress the following fact: even if the set $D_{Z}$ of the outcomes is infinite, when considered at the metalevel, it corresponds to a unique, even if not specified, element at the object level, namely the quantum state that glues the elements of $D_{Z}$ by means of the interference. Then, we have an infinite set at the metalevel and a singleton at the object evel.

### 2.2. Infinite Singletons

Conversely, let us assume that the observable is discrete and then $D_{Z}=\left\{t_{1},\cdots ,t_{m}\right\}$ is always finite. Then, $\left(\forall_{\omega }t_{n}\in D_{Z}\right)A\left(t_{n}\right)$ is simply the conjunction $A\left(t_{1}\right)\&\cdots \&A\left(t_{m}\right)$ (since the metalinguistic link *forall* coincides with the metalinguistic link *and* in the finite case). As proved in [8], the derivability of $A\left(t_{1}\right)\&\cdots \&A\left(t_{m}\right)⊢\left(\forall x\in D_{Z}\right)A\left(x\right)$ is equivalent to the validity of the formal consequence

(3) $$z\in D_{Z}⊢z=t_{1}\vee \cdots \vee z=t_{m}$$

That means: one can identify any generic element $z\in D_{Z}$ with one among the $t_{n}$ at the metalevel, but the identification is not necessary inside the formal system. We maintain the following fact: any set $D_{Z}=\left\{t_{1},\cdots ,t_{m}\right\}$, for which (3) above does not hold in the formal system, is *infinite* if considered inside the formal system—for one can count *m* elements if and only if one can distinguish those that are excluded by the failure of (3) inside the system [8].

Let us confine our attention to the spin observable. The spin can be measured for every direction *d*, and the result can be “up” ${↑}_{d}$, or “down” ${↓}_{d}$. However, spin observables with respect to different directions are incompatible: if A is prepared in state ${↓}_{d}$, then its projector has interference terms whenever a direction $d^{\prime }\neq d$ is considered.

If we prepare A in state ${↓}_{z}$ and then we measure its spin along the *z* axis, namely the observable is $\sigma_{z}$, we find outcome ${↓}_{z}$ with probability 1. In terms of formulae, this means that the set *D* of outcomes is a singleton {u}, the pure state is represented by (∀x∈{u})A(x) and the outcome of the measurement by A(u). The pure state and the outcome of the measurement are identified, or, in terms of formulae, (∀x∈{u})A(x) is equivalent to A(u). As seen above, such an equivalence means the validity of the sequent

z∈D⊢z=u

Then, we can characterize the unique element of *D* and say that *D* contains one element.

On the other side, if we prepare A in state ${↓}_{z}$ and then measure along *x* (observable $\sigma_{x}$), the result is ${↓}_{x}$ with probability 1/2 and ${↑}_{x}$ with probability 1/2. Thus, the set *D* of outcomes, counted “outside” has two elements, $D=\{t_{↓},t_{↑}\}$. The proper mixture after measurement is represented by the formula $A\left(t_{↓}\right)\&A\left(t_{↑}\right)$. The original pure state is reconstructed by the formula (∀x∈D)A(x). The two formulae are not equivalent, namely, as seen above, the following sequent, of the form (3):

$$z\in D⊢z=t_{↓}\vee z=t_{↑}$$

does not hold when describing the situation prior to measurement. We cannot distinguish the two elements of *D* prior to measurement and hence *D* is infinite inside. The vector $|{↓}_{x}\rangle$ is $1/\sqrt{2}{|↓}_{z}\rangle +1/\sqrt{2}{|↑}_{z}\rangle$ in the orthogonal basis ${|↓}_{z}\rangle$, ${|↑}_{z}\rangle$, and is often labelled |+〉, from its relative phase $+1=e^{i0}$. However, one cannot measure the phase, or, that is the same the observable “spin along *z*” is incompatible with “spin along *x*”. For this reason, a judgement of the form

z∈D⊢z=u

is not available, measuring along *x*. One cannot characterize the spin in a finite way, even if it is unique. We conclude that *D* is an *infinite singleton* prior to measurement. This is not really surprising. Algebraically, every pure state is represented as a vector |ψ〉 that corresponds to the density operator |ψ〉|ψ〉, of rank 1, even if the interference elements are not zero, that is, the projector $P_{|\psi \rangle }$ mapping |ψ〉 onto its own direction.

Clearly infinite singletons can have no extensional characterization as sets. One can provide an intensional characterization of singletons: *D* is a singleton if and only if the existential and universal quantifier coincide on it, namely

(∀x∈D)A(x)≡(∃x∈D)A(x)

for every formula *A* [8]. Then, one can easily see that, even if it is not specified (namely described by a closed term):

**Proposition** **1.** *An infinite singleton has a unique element.*

Assuming z∈D, and considering the formula A(x)≡z=x, one has (∃x∈D)z=x that in turn is equivalent to (∀x∈D)z=x by definition of an infinite singleton.

## 3. Adding Modalities

We now see how a modal operator can be introduced in the representation of quantum spins and the meaning it gets.

### 3.1. Introduction of the S4 Modal Operator

Let us consider the measurement of the spin of a certain preparation of A for the generic direction *d* and consider Equations (1) and (2). Then, we characterise the domain $D_{Z_{d}}$ for every direction *d*. For every direction *d*, the premise Γ does not contain the variable $z_{d}$ (ranging on the elements of the domain $D_{Z_{d}}$) free. The preparation is independent of the eventual outcome, for every choice of the direction. Then, Γ is *closed* with respect to all variables $z_{d}$. Moreover, the quantifier ∀ closes the formula *A* with respect to the variable $z_{d}$ for the direction *d*, and the connective $\forall_{\omega }$ does not depend on variables internal to the language. We adopt the notation □ to indicate that the formulae are closed with respect to each variable $z_{d}$ ranging on the domain $D_{Z_{d}}$ for any direction *d*. Then, Γ is □Γ and the formulae quantified by ∀ and by $\forall_{\omega }$ are all represented by □A. Then, we obtain a particular instance of a well known way to conceive the necessity modal operator as an abstraction of the universal quantifier. In the approach by basic logic, since connectives are introduced by equations, we can generalize the pair of Equations (1) and (2) to every *d*, by considering the common overall equation, written by means of □ as follows:

(4) □Γ⊢□A if and only if □Γ⊢A

The domain and the corresponding variable have disappeared, since no reference to a specific set of measurement outcomes is involved. We see that □ is the modality of the modal system S4 (we would like to remark that Equation (4) allows for deriving suitable rules of sequent calculus, introducing □ on the right and on the left, like the equations characterising other connectives, as seen in basic logic; see [9] for propositional connectives and [11] for the quantifiers. For a survey of proof theory for modal logics, see [19]. One can also observe that a similar characterization of the internal operator in lattice theory is possible).

**Proposition** **2.** *The above equivalence is valid in the modal system S4. Conversely, it entails the rules of □ in S4.*

**Proof.**  Let us assume the rules of □ in S4. From □Γ⊢A, one derives □□Γ⊢□A by *K* of S4. Then, one derives □Γ⊢□A by applying the cut rule with the premises □□Γ⊢□A and □B⊢□□B for every formula *B* of Γ. As for the other direction of the equivalence, since □Γ⊢□A and □A⊢A, one has □Γ⊢A.

Conversely, let us assume that equivalence (4) holds. Then, the necessitation rule of S4 is true assuming Γ=∅. The clause □A⊢A follows from the axiom □A⊢□A by the *only if* direction of the equivalence, and the clause □A⊢□□A follows from the same axiom by the *if* direction. Finally, in order to derive *K*, we first see that Γ⊢A entails □Γ⊢□A: assuming Γ⊢A, one derives □Γ⊢A since for every *A*□A⊢A as just seen, □Γ⊢□A follows by equivalence. Thus, since A→B,A⊢B is a provable sequent, one derives □(A→B),□A⊢□B, from which one completes the derivation of *K*. □

### 3.2. The Abstract General Projector

By the above result, the operator □ is a projector, since, from □A⊢□□A and □A⊢A, both valid in S4, one derives the idempotency of □:

□□A=□A

We can consider it an abstract form of projector, the general projector. This result agrees with the models found in quantum computation, since, as proved in [13], the logic of the process of quantum register measurement is intuitionistic propositional logic that is S4.

By definition, the general projector □ has an intermediate status, between infinite and finite, which, in particular, avoids the gap between variables and closed terms, since the formula □A associates a defined state to A, although without specifying it. Two views are therefore possible:

- □A preserves an infinite content, since □ is defined as a generalization of the quantifier ∀. We can assume that □ corresponds to an internal variable on the set of directions *d*.
- □A has a finite content, since □ is defined as a generalization of the connective $\forall_{\omega }$. We can assume that it corresponds to an external parameter.

In the infinite case, let us suppose that its equation, generalizing the equation of the quantifier, hides its own domain *T*, uniquely characterized. Then,

(5) □A≡(∀x∈T)A(x)

*T* is an infinite singleton that does not depend on any specific measurement. *T* should have a very particular status: it should contain every element *z*, not recognized, that allows for asserting *A*, in the hypothesis □Γ, namely all the unrecognized sharp outcomes of the measurement, for each direction of the spin. They should be forced to be equal, as seen in Proposition 1. In such an interpretation, the general projector □ should be considered neither positive nor negative, since every spin observable is contained in it. Indeed, if *z* is the direction of the preparation, $\sigma_{z}$ and $\sigma_{x}$ are both contained in □A. The eigenvectors of $\sigma_{z}$ are switched by $\sigma_{x}$ (and conversely): then, □A has a positive and a negative content at the same time. The paradoxical status of *T* will be clearer in the successive interpretation.

In the finite case, we need to look for a standard finite singleton, a general spin, denoted by the term ↕. It should characterise □ as a projection operator and then we could write

$$□=P_{↕}$$

adopting a notation borrowed from Hilbert spaces, even if ↕ is not a vector of $C^{2}$. Then, the formula □A would obtain a finite interpretation:

□A=A(↕)

Then, the closed term ↕ must be the witness of the necessity of *A*. Since, in S4, one has □A→A; it must witness its truth as well. Thus, □ allows for asserting that *A* is true in its finite interpretation. We can say that ↕ is *the positive term*. One should notice the difference with the $C^{2}$ couple of projectors $P_{{↑}_{z}}$ or $P_{{↓}_{z}}$, characterizing the spins ${↑}_{z}$ or ${↓}_{z}$ with some probability. Even if the value of probability is 1, probability is not truth, namely “it always happened” or “one expects it happens with certainty” is not “it happens because it must happen”, “it is forced to happen”, the certain event is not the truth. As pointed out by Qbism [20], truth is never given by a particular measurement and hence is not the simple result of experience. It can appear at a further level of abstraction, when probabilities disappear.

### 3.3. Introduction of a Temporal Parameter and Separation of Negation from Assertion

In order to reconcile truth with experience, we see how the general projector and the positive term ↕ are derived, as a particular solution of the equation introducing □, obtained when an initial condition is introduced. In this setting, one can further see how negation is separated from assertion.

In the finite interpretation, the set of all directions *d* can be ordered following a temporal parameter *t*, and an initial direction is characterized. The other directions are reached during a temporal evolution of the observable. Equivalently, the observable is fixed at $d=d_{0}$, and the initial state has a unitary evolution, described by the temporal parameter (Schrödinger picture) (actually, this is an absolutely ideal setting; one is supposed to prepare the same preparation an infinite number of times and apply, each time, the corresponding observable, or to prepare an infinite number of times the initial state, make it evolve to the state corresponding to the temporal parameter, and apply the fixed observable). In practice, the fixed observable is applied (mentally) to the points of the surface of the Bloch sphere rather than to the initial point only. In the following, we shall apply the Schödinger picture. We are interested in two cases: when the direction *d* is the same of the initial preparation, *z* that is the observable is $\sigma_{z}$, represented by the Pauli matrix $\sigma_{Z}$, and when the observable is $\sigma_{x}$, represented by the Pauli matrix $\sigma_{X}$ in the computational basis (direction *z*).

Let us consider $\sigma_{z}$ and then a generic assertion of the form

(6) $$□\Gamma ,\sigma_{z}⊢A\left(t\right)\;\;forall\;\;t\geq t_{0}$$

where the initial observable is put as a hypothesis, the temporal parameter *t* and the initial time $t_{0}$ are specified. We need to find its sum over time.

The eigenstates of $\sigma_{z}$ are found with probability 1 and, by the Born rule, any different state obtained during the temporal evolution is found with probabilities given by the squared modulus of the probability amplitudes of the state itself. We maintain that $\sigma_{z}$ “tells the truth” since it “*always* tells the truth” on the state of the particle, namely truth is independent of probability when probability is considered for each point of the surface of the Bloch sphere. Then, the truth on the state of the initial point is established, even if it can be identified with the explicit result of experience only when the initial point is one of the two poles. Hence, we identify the sum over time of the assertions (6) above with the finite version of □, namely we put:

(7) $$□\Gamma ⊢□A\;\equiv \;□\Gamma ,\sigma_{z}⊢A\left(t\right)\;\;forall\;\;t\geq t_{0}$$

where □ has the finite interpretation only. The observable $\sigma_{z}$, in the basis ${↑}_{z}{,↓}_{z}$, is represented by the diagonal Pauli matrix $\sigma_{Z}$ that can be read as the sum of the two projection operators $P_{{↑}_{z}}$ and $P_{{↓}_{z}}$:

$$\sigma_{Z}\equiv \left(\begin{array}{cc} 1 & 0\\ 0 & -1 \end{array}\right)=\left(\begin{array}{cc} 1 & 0\\ 0 & 0 \end{array}\right)-\left(\begin{array}{cc} 0 & 0\\ 0 & 1 \end{array}\right)\equiv P_{{↑}_{z}}-P_{{↓}_{z}}$$

Introducing the positive term ↕ to describe the finite version of □, that is, the general projector, as $P_{↕}$, $\sigma_{z}$ is described by means of one projection operator instead of two. This confirms that □ can abstract the projectors.

We would like to briefly recall a view considering “internal measurements”, introduced in [21], to the aim of tuning our reasoning with the internal logic of quantum computation. In such a view, the result of a measurement with observable $\sigma_{z}$, namely what is seen by an observer who is obviously external to the quantum black box, is in accordance with the hypothetical “internal observer” performing internal measurements (see [22]; an overall treatment is contained in [23]).

The second case is to measure the spin with respect to the *x* direction, while the other assumptions are the same: the same preparation and temporal evolution. Then, one has a generic assertion of the form

$$□\Gamma ,\sigma_{x}⊢A\left(t\right);\;forall\;\;t\geq t_{0}$$

It is summarised by an operator ${□}_{n}$, implicitely defined putting the equation

(8) $$□\Gamma ⊢{□}_{n}A\;\equiv \;□\Gamma ,\sigma_{x}⊢A\left(t\right)\;\;forall\;\;t\geq t_{0}$$

Let us assume that the equation has its own solution and reason as in the previous case to see its features. In the basis ${↑}_{z}{,↓}_{z}$, the observable $\sigma_{x}$ is represented by the off-diagonal real Pauli matrix $\sigma_{X}$, which is the sum of the two antiprojectors $Q_{{↑}_{z}}$ and $Q_{{↓}_{z}}$:

$$\sigma_{X}\equiv \left(\begin{array}{cc} 0 & 1\\ 1 & 0 \end{array}\right)=\left(\begin{array}{cc} 0 & 0\\ 1 & 0 \end{array}\right)+\left(\begin{array}{cc} 0 & 1\\ 0 & 0 \end{array}\right)\equiv Q_{{↑}_{z}}+Q_{{↓}_{z}}$$

where $Q_{{↑}_{z}}$ transforms ${↑}_{z}$ into ${↓}_{z}$, and $Q_{{↓}_{z}}$ conversely. Then, ${□}_{n}$ should be conceived as a *general antiprojector*. Notice that, since the preparation is fixed, ${□}_{n}$ is not something dual to □; it is rather a derivative of it, due to the shift between the preparation and the observable. The antiprojector arises as a solution of the above particular Equation (8), hence it has no infinite description analogous to (5). As for its finite description, since □ is $P_{↕}$, ${□}_{n}$ is $Q_{↕}$ that generalizes $Q_{{↑}_{z}}$ and $Q_{{↓}_{z}}$. The result of $Q_{{↑}_{z}}$ applied to ${↑}_{z}$ is ${↓}_{z}$, which is the only element of $C^{2}$ that is provably different from ${↑}_{z}$, since, in quantum mechanics, two vectors can be distinguished with certainty if and only if they are orthogonal, analogously for $Q_{{↓}_{z}}$. Then, an antiprojector implies the existence of another, different, element. Thus, we introduce a term ${↕}_{n}$, characterizing the result of $Q_{↕}$ that is conceived as “the element different from ↕”. It is the *negative term*. There is a flip between the positive and the negative term, analogous to the flip determined by the matrix $\sigma_{X}$ on the basis of the Hilbert space. Then,

$${□}_{n}A=A\left({↕}_{n}\right)$$

where ${↕}_{n}$ is characterized by

$${z=↕}_{n}\;\equiv \;z\neq ↕$$

One can interpret ${↕}_{n}$ as a negative element that, attributed to *A*, is anyway a witness of *A*. It is like an assertive form of negation. Then, ${↕}_{n}$ is found as an odd element of the infinite singleton *T*, besides its standard element ↕. For quantum states, namely infinite singletons, the characterisation of two opposite elements ↑ and ↓, in which an infinite singleton unfolds, is bearable, by means of probabilities. Let us see that it is not bearable when probabilities disappear, namely with the modality: it implies the arising of the non contradiction law.

### 3.4. Modal Uncertainty and Modal Negation

We first observe that, since the mixed state described by the density operator $\frac{1}{2}P_{↑}+\frac{1}{2}P_{↓}$ does not depend on the direction *d* considered for the spin observable, it can be described by a constant of the logical language, let us denote it by ⊥. Being ⊥ constant with respect to the variation of *d*, by definition ⊥=□⊥. Then, we extend the meaning of ⊥ to the pair ${↕,↕}_{n}$, found by abstraction, for which no notation in Hilbert spaces is available. $\sigma_{z}$ and $\sigma_{x}$ are incompatible observables. Let us consider their “sums over time”, namely the operators □ and ${□}_{n}$ found by abstraction. Since the pair $□,{□}_{n}$, *yields* the pair ${↕,↕}_{n}$, one can try to transfer the uncertainty to logic, writing the following *modal uncertainty*:

(9) $$□A,{□}_{n}A⊢⊥$$

which is interpretable as a contradiction between the modal formulae □A and ${□}_{n}A$. Let us see that modal uncertainty is consistent with the standard view of contradiction.

**Proposition** **3.** *Modal uncertainty is equivalent to an instance of the non contradiction law.*

**Proof.**  Let us write □A in its finite form A(↕) and rewrite ${□}_{n}A$ as $A({↕}_{n})$. Thus, modal uncertainty is rewritten $A\left(↕\right),A\left({↕}_{n}\right)⊢⊥$. On the other side, as seen above, in its infinite form, □A is (∀x∈T)A(x), where *T* is unique and is an infinite singleton. Then, let us assume both A(↕) and $A({↕}_{n})$. Then, if *z* is the generic unique element of *T*, one has both z=↕ and ${z=↕}_{n}$ (we can adopt the same letter *z* since any two unspecified elements chosen in an infinite singleton can be proved to be equal, as implied by the definition of infinite singleton, see Proposition 1). Since ${z=↕}_{n}$ means z≠↕, one finally has the equivalent writing:

z=↕,z≠↕⊢⊥

which is an instance of non contradiction, expressed under the form of the law of identity.

It is important to notice that, in order to see the above equivalence, one has to consider both the infinite and the finite point of view, and exploit the assertive form of negation.

Then, let us define a negation ∼ on modal formulae, *modal negation*, putting:

(10) $$∼□A\equiv {□}_{n}A$$

Modal negation, by definition, satisfies the non contradiction law. Applying □, which has also an infinite character, yields the finite operator ${□}_{n}$. For this reason, the converse definition, namely putting $∼{□}_{n}A\equiv □A$, is not correct: modal negation is not bivalent, since one cannot recover the infinite. As a consequence, like intuitionistic negation, modal negation cannot satisfy the excluded middle law. It should be noticed that, since the uncertainty is interpretable as non-contradiction by Proposition 3 and hence it results in negation as in definition (10) above, the overall effect of modal negation, which is the general antiprojector, is to lie with respect to the result of the general projector. Then, when applied to an eigenvector, it answers the opposite eigenvector.

We finally notice that a bivalent negation can be found putting:

$$∼A\left(↕\right)\equiv A\left({↕}_{n}\right)\;\mathrm{and}\;\mathrm{conversely}\;∼A\left({↕}_{n}\right)\equiv A\left(↕\right).$$

Then, since $A({↕}_{n})$ is ${□}_{n}A$, one has

(11) A(↕)=∼∼□A

In conclusion, negation is a finitization, and double negation finds the finite content of □A. Such a conclusion is consistent with well-known logical results, concerning the double negation translation of classical into intuitionistic logic [24] and the equivalence of classical and intuitionistic logic to derive negated formulae that follows from Glivenko’s theorem [25]. We recall in particular that the following definition, on the basis of propositional classical negation ∼ and the modal operator □, given by Gödel [12]:

¬A≡∼□A

finds intuitionistic negation. We leave to further work to see how intuitionistic logic could be found from infinite singletons rather than from the pair of opposites sharp values of propositional classical logic, namely, to see how intuitionistic logic can be recovered from qubits, as proved in [13] with different techniques, rather than from bits, as in the original proposal by Gödel. □

## 4. Freudian Theory

Now, we will try to use the instruments hence developed in order to put forward a novel interpretation on the mind architecture, by drawing on some Freudian and post-Freudian (briefly, psychodynamic) ideas on psychic functioning. Since the amount of changes these ideas endured already during Freud’s life and their subsequent diffusion into the wider scientific discourse, often in diluted if not plain wrong forms, we shall seek to define the notions we employ as precisely as possible, as well as providing some historical and epistemological cues necessary for their correct interpretation.

Namely, the foundations of psychodynamic model of mental apparatus are deeply embedded in Freud’s early neurological works, especially in his book On Aphasia [5]. There, he postulated a model (which we could, a posteriori, classify as *connectionist*) of the mind–brain relations based on the idea of *mental representation*. Synthetically, mental representation is a sort of a psychic delegate of neural changes, and it allows for the establishment of the psychic super-structure of the human being. That is, mental representation can be considered the foundational unit of the psychic order. The subsequent distinction of two different types of mental representations, namely the *thing-* and the *word-presentations*, makes their role a bit clearer. The former is the set of impressions, both sensory and motor ones, related to an object (which can be either internal or external), while the latter is a complex association of sensory and motor elements which define a single word. In Freud’s view, word …*acquires its meaning by being linked to a thing-presentations, and not by reference to the thing itself* [5] (p. 213) Therefore, this entails that the symbolic function of the mind depends upon the establishment of the associations between thing-presentations and the corresponding word-presentations. This statement was taken as the basis of the clinical practice of psychoanalysis, but its theoretical power has been somewhat underestimated.

Additionally, Freud further elaborated this point in his later works and specified that not only the aforementioned two types of mental representations pertain to different domains of psychic activity, but they also define two different mental systems—namely the Conscious and the Unconscious:

*The conscious presentation comprises the presentation of the thing plus the presentation of the word belonging to it, while the unconscious presentation is the presentation of the thing alone.* [26] (p. 201)

It is worth noticing here that, with his idea of presentation, Freud suggests an approach to the initial issue of this paper, namely: the relation between language and the characterization of objects. His approach is somewhat unique even in an epistemological perspective, and hence allows for addressing such an issue in new ways. The Freudian view concerns the *structure* of representations, namely, as originally underlined by Freud himself, thing presentations are open and word presentations are closed. These differences stem from their origins and different mental habitats, but we must highlight that the idea of openness of the representations closely mirrors Frege’s conception of terms of first order language [27,28], as we have previously shown in [29]. In the next section, we shall see that the idea of characterization of objects via infinite singletons proposed in the present paper accomplishes the structure of Freudian representations.

These considerations allow us to introduce and discuss the concept of *Unconscious*, surely the most widely known intellectual heritage of psychoanalysis. However, the idea of Unconscious still represents an important stumbling block on the path of integration of psychodynamic knowledge and other scientific disciplines (especially the so-called “hard sciences”), as well as one of the more complex Freudian ideas. For the sake of clarity, we will try to pinpoint its meaning in two steps. First, we must distinguish the psychodynamic conception of Unconscious from the ones found in cognitive psychology or neurosciences, while cognitive theories usually limit the definition of unconscious processes to instances of unconscious cognition such as subliminal perception and implicit memories, in neurosciences, the focus in on our lack of awareness of neuropsychological processes underpinning our conscious experience (such as neurons firing, activation of different areas of cortex, etc.). A relevant attempt to bridge the gap between the psychoanalytic view of conscious and unconscious processes and the neurosciences has been recently proposed by [30], who built on the statistical mechanic principle of minimization of the free energy in order to show that the Freudian theory of the primary process [6] is coherent with the functional organization of the cortical neural activities; a different model in the same line of research was shortly later proposed by [31], who explored possible neural substrate of the Freudian Ego functions. These studies document the coherence of the Freudian theory of the conscious and unconscious processes to the micro level of neural dynamics.

On the other hand, the inner coherence of the Freudian theory can also be documented by abstraction, that is, by making the effort to describe in a formalized way the structural constraints of the unconscious representations; as abstract theories have an higher degree of generality, we expect that the formal theory will be more suitable than the descriptive one exploiting its explanatory power.

In order to understand how the conception of the Unconscious differs from the previous ones according to the psychodynamic view, we can start by tracing its historical and epistemological origins. Namely, the Unconscious is a concept *first encountered in a clinical situation* [32] (p. 128), where it stands as an *explanans* for the different instances of otherwise inexplicable psychic acts and behaviors. That is, without assuming the existence of an unconscious mind, the scientists and the practitioners are left without adequate tools for understanding of frequent gaps in consciousness and a seeming lack of sense of some behaviors. Therefore, we can positively *describe the Unconscious as a concept whose justification lies in its ability to constitute and organize a new field of objectivity and intelligibility* ([33], cited in [32], p. 128).

Second, to further delineate the conceptual borders of the Unconscious as used here, we must distinguish between different psychodynamic conceptions of the Unconscious. The most widely accepted one is based on the idea of *repressed content* such as thoughts, impulses, and affects, which are inadmissible for the conscious self-image of the individual and therefore removed. This idea informed the early view of psychodynamic practice as uncovering of the repressed, of overcoming resistance and becoming aware of one’s admittedly unacceptable wishes and repressed memories. However, the view we subscribe to in this paper has roots in the aforementioned structural contraposition of the *linguistic Consciousness and wordless Unconscious* [32] (p. 131) and sees the Unconscious as a different type of mentation, comparable to the conscious one, in line with the first Freudian formulation of a theory of the Unconscious, the one originally proposed in The Interpretation of Dreams [6].

This theoretical position therefore defines the Unconscious not in terms of its content but in terms of its structure. Thus, the idea of structural Unconscious implies that any model of the mind must include a separate ideation, *a different thought constructing agency* [34]. The introduction of unconscious ideation as an equitable peer of the conscious one also requires a description of its organizing principles. The most notable such description is found in the book The Interpretation of Dreams [6], where Freud characterized Unconscious as akin to the so-called the primary process. Namely, while the aforementioned absence of words from the Unconscious remains its most distinctive characteristic, its non-discursive nature is outlined by the following set of principles:

- Displacement;
- Condensation;
- Absence of contradiction;
- Substitution of the external reality with the internal one;
- Timelessness.

It is important to consider that, in Freud’s view, the two conceptions of the Unconscious (the structural one and the repressed one) are not contradictory as the repressed representations are exactly found to be treated in accordance with the laws of the structural Unconscious. These principles were later defined as *laws of imagination* by Langer [35] and hypothesized as a moving force of all art forms, among else. Likewise, these laws underline the fact that Unconscious thoughts are formed using particular methods of construction, association and synthesis, which are different from those of the conscious mind. For instance, the first two points refer to the tendency of the Unconscious to condense apparently different, even opposite, items of information and subsequently to its capability of seamlessly treating such peculiar mental objects. Subsequently, since the opposites condense together, there is no negation in the Unconscious, and hence the law of non-contradiction does not hold. While this original description relied on the elements of energetic, dynamic, and logical perspectives, the latter point was subsequently emphasized by Freud, underlying the fact that Unconscious creates its own logical environment, where one finds condensation and displacement rather than the usual logical connectives, in order to process the information. In his words, *The governing rules of logic carry no weight in the Unconscious; it might be called the Realm of the Illogical* [36] (p. 168).

However, this important path opened by consideration of logical features of unconscious ideation remained almost entirely unexplored in the subsequent development of the psychodynamic theories. The Hungarian psychoanalyst Imre Herman [37] proposed the idea that the deep Unconscious can only be described in logical terms, while Arieti made an interesting comparison *between the Aristotelian logic of conscious thought and the paleologic of primary process thought* ([38], cited in [32], p. 131). It was not until the Chilean psychoanalyst I. Matte Blanco [7] that this aspect of Freudian thought received serious consideration. He proposed a reformulation of Freudian Unconscious in purely logical terms [7]. Namely, he described the Unconscious as a standalone way of thinking and being, denominated *Symmetric Mode* and characterized by two principles:

- the Principle of Symmetry;
- the Principle of Generalization.

According to Matte Blanco, *the Unconscious Mode treats the asymmetrical relations as if they were symmetrical* [7] (p. 38). The word symmetry refers to the sameness, identity between two things, and their fundamental indistinguishability. In particular, since the relation of contradiction is, nevertheless, a relation, the Unconscious treats opposites as identical. Furthermore, since the time can be described as a series of moments where one follows the other, one bizarre consequence of the application of the principle of symmetry is the complete disappearance of time. On the other hand, the Principle of Generalization reflects the fact that Unconscious does not deal with individual elements, but only with classes to which they belong. To provide an example, for the child, the mother is not a single, individual person, it is rather a sum of all of the attributes of all members of its defining class—the class of mothers. Therefore, the individual thing is made identical to the class it belongs to. Based on Dedekind’s observation that, if a set is equivalent to its part, the set itself is necessarily infinite [39], Matte Blanco’s formal explanation accounts for the infinite, all-or-nothing character of Unconscious processes.

We need to stress that, subscribing to a perspective suggested by both Freud and Matte Blanco, the primitive roots of thinking are unconscious and hence the original mode of thinking is infinite. Likewise, consistently with what has been proved in Section 3.3 and clarified by Definition 10, we have shown that it is useful to reason considering finite as the negation of infinite and not the other way around. In a way, language points us in a wrong way since the kind of infinite we are familiar with—the mathematical infinite—is only a derivative of the original symmetric infinite, and can only be conceived after the finitization of single elements.

## 5. Back to Infinite Singletons and Modalities

Following *The Unconscious as Infinite Sets*, the guidelines for finding a logical embedding of the Symmetric Mode into the usual logical setting are provided by the following assumptions:

- every set is infinite;
- every relation is symmetric.

Since relations are all symmetric only in singletons (for, if a,b∈U, and a≠b, one can put a<b), the solution is to have *infinite singletons* [40], which can account for the symmetry and the generalisation principles of Matte Blanco’s Bi-logic.

We further observe that infinite singletons can accomplish Freud’s original requirement on presentations as well. Given a set *D*, and assuming its characterisation as an infinite singleton in terms of quantifiers, namely: for every formula *A*

(∀x∈D)A(x)≡(∃x∈D)A(x).

One asserts, on one side that *D* is non-empty, since the usual way to conceive the existential formula (∃x∈D)A(x) hides a conjunction: ∃x(x∈D&A(x)). Then, some object is denoted. On the other side, the usual way to conceive the universal formula (∀x∈D)A(x) is hypothetical: ∀x(x∈D→A(x)). This means that new undefined elements can be added to the existing undefined element, namely, the representation is open, even if its denotes a closed ambitus that of the undefined represented object indeed. Uniqueness of the object is guaranteed by Proposition 1. The quantifier is a way of closing without closing. When the characterisation of the object is linguistically added, denoting it by a closed term of the language, *u*, both the existential and the universal formula are equivalent to the propositional formula A(u). Then, word presentation is reached and nothing more can be added. Since standard singletons satisfy the characterisation of infinite singletons, Freud’s requirement (*The conscious presentation comprises the presentation of the thing plus the presentation of the word belonging to it, while the unconscious presentation is the presentation of the thing alone* [26] (p. 201)) is satisfied, together with the corresponding separation of thing and word presentations as open and closed, respectively, as observed by Freud as well.

We have seen that infinite singletons positively describe the logical features of characteristic unconscious processes such as condensation and displacement [17]. Furthermore, we can say that access to consciousness is guaranteed by time, which is introduced in the system through the contact with external reality [17]. As stated,

*The pivotal consequence of this model is that the unconscious elements cannot be characterised in the absence of external reality, which produces the collapse of infinite sets and allows for the emergence of linguistic representations.* [17] (p. 46)

Afterwards, we can see that the Freudian characterization of the mental processes, both conscious and unconscious ones, corresponds to the representations obtained for quantum spins as previously described. To this aim, we need to consider absence of time, negation, and non contradiction.

In Section 3.3, we have shown that the introduction of time as an external parameter allows us to subsequently introduce, by Equations (7) and (8), modal assertion and modal negation. Then, we witness the emergence of the law of non-contradiction, as proved in Proposition 3, and, consequently, we define the negation via (10). Then, the secondary process, which takes into account external reality and hence finite elements, becomes possible. In this scenario, the aforementioned modal operators behave like mediators between the primary and secondary processes. To illustrate the role of modalities in achieving the finitization of infinite singletons, we would like to reconsider an example originally proposed by Freud.

### Negation in Freud

As we have seen, infinite singletons are not extensional, i.e., they are objects with no associated name. This mirrors Freud’s original description of the general requirement for the access to the conscious mind via a link to word-presentations. In Matte Blanco’s words, this entails finitization. Therefore, in the modal interpretation of quantum spins, finitization comes together with negation; see definition (10) and equality (11), which is consistent with the Freud’s observations concerning negation, expressed in the homonymous paper from 1915 [18]. In fact, Freud considers negation to be a *de-fusion*:

*The general wish to negate, the negativism which is displayed by some psychotics, is probably to be regarded as a sign of defusion of instincts that has taken place through the withdrawal of the libidinal components.* [18] (p. 235)

Following Freud, negation is *the intellectual counterpart of repression* and therefore the end of the infinite mode. The example from the paper is as follows: a patient tells his dream, in which an unspecified person is contained, to the psychotherapist. When asked:

*Who was that person?*

the patient answers

*It is not clear, but for sure she was not my mother.*

The psychotherapist can conclude that the person was in fact the patient’s mother. Freud’s explanation is based on the idea that the patient had repressed that fact and hence he denies it rather than admitting: negation is the symptom of repression. In our terms, we can formalise the whole process as follows: The patient had an original conscious information about his mother, characterised by the sharp state z=mother. Then, he has repressed this and hence that information is contained only in the Unconscious. Particularly, since it is has been subjected to the laws of Unconscious, it exists in the superposed state − mother + not-mother. Then, he has repressed this. Hence, that information is contained only in the Unconscious, in the superposed state mother + not-mother, since condensation with the opposite occurs in the Unconscious. This means that a Hadamard gate has been applied to the original information. Afterwards, the new state is processed in the dream. When asked to characterize the person, the patient should correctly apply the observable $\sigma_{z}$, in order to judge, and he actually does. It is important to consider that, assuming Freudian theory, any mental function, including judgement, must consider the Unconscious. Then, in our view, the whole Bloch sphere, not only its north and south poles, are considered by the function of judgement. This means that any judgement really follows from the application of the modality rather than from a single measurement (one could further explore the psychological implications of this point, which could explain why there are phenomena such as the normative value of reality, the prescriptive role of examples, the need to do the opposite as a reaction to authority, and so on). Had the patient not repressed, the final answer would have been the eigenvector “my mother”. Since a Hadamard has been applied to the eigenvector, “I don’t know” becomes the first correct answer actually verbalizable, obtained by the application of $\sigma_{z}$. However, the patient is asked to characterize the person. Then, he adopts the observable for which the actual state of his information is an eigenvector that is $\sigma_{x}$. This means that he is performing a judgement by means of the negative-dominated Equation (8) whose solution is the negative modality ${□}_{n}$ that creates the negation connective, as we have seen in definition 10. Then, his second correct answer, the word presentation of his mind’s content, is: “not my mother”. In Freud’s words:

*The performance of the function of judgement is not made possible until the creation of the symbol of negation has endowed thinking with a first measure of freedom from the consequences of repression, and, with it, from the compulsion of the pleasure principle.* [18] (p. 239)

## 6. Perspectives and Future Work

New perspectives for the interpretation of the meanings of quantum entities have been opened by the arising of Quantum Information [41]. We think that our line of research is close to some recent approaches, mainly developed outside logic. Recent research has addressed issues stemming from “Wigner’s friend” thought experiment, considering different levels of judgements (see [42,43], where the role of time is also considered). We further quote [21], where different levels of judgements are conceived adopting a hypothetical “internal observer” rather than a “super observer” (see Section 3.3). Finally, QBism has formulated a quite radical view, reconsidering Bohr’s interpretation and making a proposal in terms of an epistemic and subjective view of probabilities [20] that allows for distinguishing between probability one and truth, as also discussed by our approach (for a recent discussion on QBism, see [44]).

Our approach is a foundational construction that can lead directly to applications in different fields of psychoanalysis. As for the foundations, it deals with the issue of distinguishability and other related ones, including the measurement problem, which have intrigued quantum physics since the beginning [45] and have subsequently led to the modal interpretations, the first of which is proposed in [46]. The development perspectives of our approach, for theoretical physics, rely also on the fact that it can show how the representation of quantum objects corresponds to mental representations, by which unavoidably we interpret nature. With this respect, our approach that carries infinitary constructions is fully consistent with the side of the mental representation of reality, since our mind starts from the infinite [7]. Consistently with Matte Blanco’s view, the suggestion is that there is an infinite primary mode that can actually consider indefinite objects, from which logic itself can emerge.

As for the applications to psychoanalysis, developing formal models can open new perspectives to the modelization of psychoanalytic theory itself, since it can offer a novel conceptual platform for the consideration of different theories and observations. Specifically, development of formal models in psychoanalysis is a recent research topic which has put in contact clinical and computational research (see [47,48,49] for quantum models). Moreover, the introduction of quantum models in the domain of cognitive research gave birth to a new field termed “Quantum Cognition”. However, in the light of recent findings, including ours, we would like to stress that useful modelling of the mind should avail of the functions of what is usually termed “dynamical Unconscious” for its cognitive activity as well. In other words, why should cognition be separated from affects? We should aim at “Quantum affective cognition”, mirroring Vigotsky’s idea that *there is a hidden affect behind every thought*. We would like to quote the very recent proposal described in [50,51], for which *quantum theory reflects fundamental principles for human cognition*. It adopts the Bloch sphere as an indispensable tool for semantics, since it allows for the *integration of information*, proposing a model of subjective task-oriented meaning built on the model of emotions and hence integrating affects. Note that, in the Bloch sphere, the undefined phase component captures the indefinite and infinite aspect of affective cognition, which is represented in our model by the infinite singleton. Therefore, both models support the relevance of the quantum formalism in the modelisation of the human mind. Finally, we must note that efforts invested into development of the present model, that is based on the analysis of language, are also motivated by the need to keep a close contact with the clinical side [17,52].

Regarding the future research paths, we believe that additional techniques capable of importing the indefinite into logic could be explored. Then, the nexus between the idea of infinite singleton and the *word-* and *thing-presentation* could be examined with a greater acumen. Afterwards, we propose a further development of the logic of modal formulae presented here, to include the normative aspects of psychoanalytic theory considered in the second topic. One particular issue needing further study is related to the relations of the modalities with intuitionistic logic (recently, a theoretical suggestion considering unconscious processes in terms of intuitionistic logic has been proposed in [53]). On a different note, since a clear correspondence between the modalities of *S*4 and the exponentials of linear logic [54] has been proved in [55], future research should consider linear logic as well. In previous works on the representation of quantum states in first order language [8], the application of the multiplicative connectives of linear logic has been considered in modeling multi-particle quantum systems. This could represent another potentially fruitful research trajectory. Finally, in order to discuss the relationship with the aforementioned logical systems, a possible technical development would require a suitable extension of the equations introducing the modal operator into the multiplicative case. To this aim, the role of the identity in the formulation of the structural rules of sequent calculus and of the exponentials of linear logic should be taken into account. 

## Data Availability

Not applicable.
